# Modulated electrochemical force microscopy: Investigation of sodium‐ion transport at hard carbon composite anodes

**DOI:** 10.1111/jmi.13417

**Published:** 2025-05-09

**Authors:** Sven Daboss, Nikolas Franke, Beatrice Fraboni, Christine Kranz, Tobias Cramer

**Affiliations:** ^1^ Institute of Analytical and Bioanalytical Chemistry (IABC) Ulm University Ulm Germany; ^2^ Department of Physics and Astronomy University of Bologna Bologna Italy

**Keywords:** atomic force microscopy, hard carbon, electrochemical strain, sodium ion batteries

## Abstract

For sodium (Na)‐ion batteries (SIBs), the next generation of sustainable batteries, hard carbon (HC) composite electrodes are the most used anodes. Here, we demonstrate the potential of modulated electrochemical force microscopy (mec‐AFM) to investigate electrochemical strain due to ion insertion at the electrolyte/electrode interface. HC composite anodes have a complex, multiphase structure, which include the HC particles, conductive carbon nanoparticles (carbon black) and the binder. To address the effect of the composite material on the sodium‐ion transport, we employ mec‐AFM. A HC composite anode was embedded in an epoxy‐polymer matrix and was polished to expose a micro‐sized area that enabled high‐frequency modulation of the ion transport. We analyse the influence of the modulation on interfacial forces and its role in generating electrochemical strain in the composite anode. Multichannel mec‐AFM imaging at varying electrode potentials revealed that the observed electrochemical strain predominantly occurred in the softer binder matrix rather than in the HC microparticles. Our findings underscore the significance of ionic transport pathways through the binder matrix and establish mec‐AFM as a novel AFM‐derived technique for visualising ion dynamics at battery interfaces.

## 1 INTRODUCTION

The global transition toward sustainable energy storage is the driving force for the development of next‐generation rechargeable batteries. A key aspect of advancing these post‐lithium batteries is understanding the ion transport mechanism and the complex processes at electrode–electrolyte interfaces, which among other aspects, influence the electrochemical stability, the cycle life, the long‐term performance, and the solid‐electrolyte interphase (SEI) formation.^1^, ^2^ Although, lithium‐ion batteries (LIBs) are still dominantly used in portable electronics and electric vehicles, given their high energy density and long cycle life,^3^, ^4^, ^5^ sodium‐ion batteries (SIBs) are currently considered as alternatives for rechargeable energy storage primarily due to the abundance of sodium, low cost and higher safety.^6^, ^7^ Within recent years, suitable anode and cathode materials have been developed for SIBs, with carbon‐based composite electrodes as anodes for the development of sustainable and efficient batteries.^8^, ^9^, ^10^ The larger radius of sodium (Na)‐ions compared to lithium (Li)‐ions, makes intercalation in graphite anodes, mainly used in LIBs a challenge, for example, resulting in limited reversible capacities.^10^, ^11^, ^12^, ^13^

Hard carbon (HC) has emerged as viable anode material for SIBs due to its high capacity, structural tunability, low operating voltage and excellent reversible specific capacity.^14^, ^15^, ^16^ HC consists of sp^2^ and sp^3^ hybridised carbon domains with a porous structure that is influenced by the pyrolysis conditions and precursor structure.^9^ The presence of nanopores, heteroatom containing defect sites and curved layer stacks with variable d‐stacking distances are crucial for the reversible insertion of Na‐ions.^16^ HC composite electrodes for SIBs consist typically of HC particles, conductive carbon nanoparticles and a binder in a ratio of 85%:10%:5%.^17^, ^18^ As the Na‐ion transport within the electrolyte and at the cathode and anode plays a crucial role, in situ techniques capable of investigating ion transport processes are important to understand the factors governing the ion transport and with that the performance of SIBs.

Ion transport in battery electrodes and electrolytes has been studied via electrochemical techniques such as electrochemical impedance spectroscopy (EIS)^19^ or galvanic intermittent titration technique (GITT).^20^ Ledwoch et al. used GITT in combination with other techniques such as electrochemical potential spectroscopy (EPS) to elucidate transport phenomena of Na‐ions in HC anodes and to determine the diffusion coefficients.^21^ Xu et al. revealed Li‐ion transport and local phase changes at Ag_1.66_Mn_8_O_16_ cathodes using in situ transmission electron microscopy (TEM), electron diffraction, spectroscopy, and theoretical simulations.^22^ Lee et al. determined the influence of different electrolytes on the Na‐ion transport in HC composite anodes by using EIS to analyse the charge transfer resistance and GITT to measure Na‐ion diffusion coefficients.^23^

Mechanical strain and stress during sodiation and desodiation of HC composite electrodes is directly related to ion transport. The investigation of mechanical stress is relevant, as it potentially leads to degradation effects such as cracking, delamination, and structural changes, which have been subject of several studies. Chanda et al. measured the stress evolution in HC composite anodes studying the effect of different binders (sodium carboxymethyl cellulose (CMC), styrene butadiene rubber (SBR), polyvinylidene fluoride (PVDF)) during electrochemical cycling using the substrate‐curvature method.^24^ The studies have revealed a correlation between induced stress and the electrochemical reactions, with a compressive stress of –5.9 to –6.8 MPa during sodiation due to Na^+^ insertion and volume expansion, followed by tensile stress (1.5–3.3 MPa) during desodiation as Na^+^ was removed. Also X‐ray computed tomography (XCT) has been used to examine structural changes in HC composite electrodes during sodiation and desodiation to visualise volume changes, tortuosity, and the Na‐ion concentration distribution.^25^ *Operando* X‐ray diffraction (XRD) of HC electrodes during desodiation enabled real‐time monitoring of phase transitions and structural changes.^26^ *Operando* TEM studies have shown the sodium insertion and volume expansion occurring during different charging stages with nanoscale resolution.^27^ Particular differences are observed between the initial sloping phase, during which ion intercalation is strongly potential dependent and the plateau region, where insertion occurs close to the Na/Na^+^ redox potential.^28^ Such a multistep model has also been confirmed by *operando* nuclear magnetic resonance (NMR) studies showing that during the final stage of the insertion process, nanopores of the HC particles are filled with Na clusters with metallic character.^29^, ^30^

Scanning probe microscopy (SPM) techniques offer an alternative approach to resolve interfacial transport phenomena in battery electrodes, but they are less frequently reported in the literature. Schougaard and co‐workers presented scanning electrochemical microscopy (SECM) and scanning ion conductance microscopy (SICM) to investigate the Li‐ion transport in lithium titanate (Li_4_Ti_5_O_12_, LTO).^31^ Scanning electrochemical cell microscopy (SECCM) was used to map spatial heterogeneities in the electrochemistry of active cathode material, for example, LiFePO₄ electrodes, linking these variations to local structural properties.^32^ SECCM was also used to visualise local Li⁺ transport in LiFePO_4_ composite electrodes and single particles, providing insights into ion transport mechanisms at the microscale. Unwin and coworkers investigated SEI formation on highly oriented pyrolytic graphite (HOPG), demonstrating that step edges influence the SEI formation, with step edges fostering a more passivating and stable SEI.^33^

**FIGURE 1:**
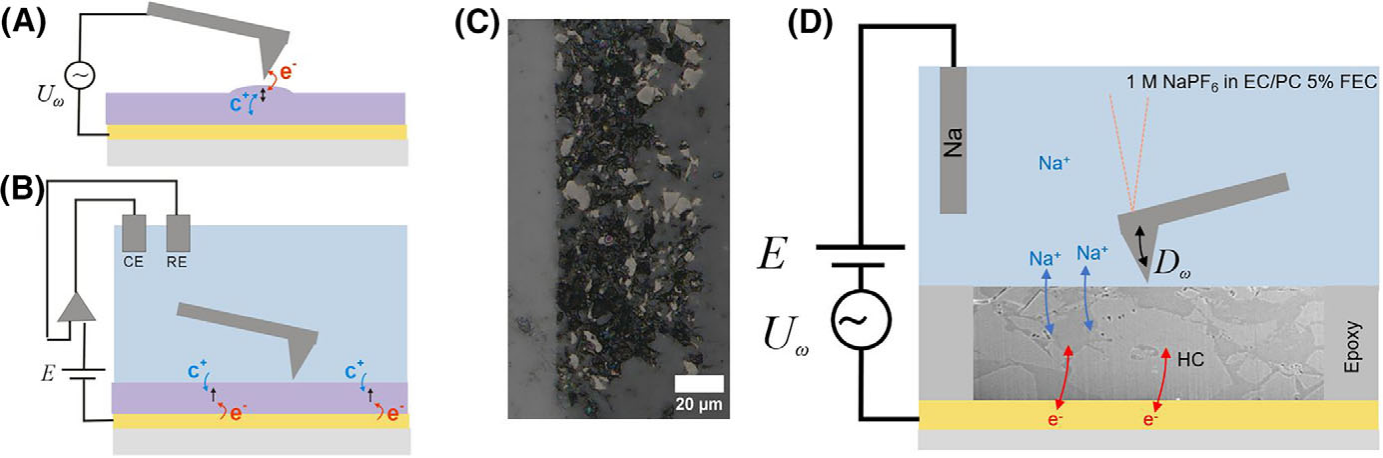
Scheme of AFM techniques and measured sample. (A) ESM: local tip sample interactions induce oxidation and reduction combined with ionic displacement currents below the AFM tip at the frequency of the applied AC signal *U_ω_*. (B) ec‐AFM: AFM tip monitors morphological changes caused by electrode oxidation/reduction processes and ion insertion at the biased substrate. (C) Optical microscopy image of the epoxy embedded and polished HC electrode surface, showing the distribution of HC particles (particles in white). (D) Scheme of mec‐AFM: an AC modulation is integrated into the control electronics, charging the micro‐sized electrode at the applied AC bias frequency. The AFM cantilever picks up surface deflections *D_ω_* generated by the electrochemical strain. The composite HC microelectrode is represented by a FIB‐SEM cross section image (see also Figure S2).

Atomic force microscopy (AFM) and AFM‐based techniques such as AFM force spectroscopy, conductive AFM (c‐AFM), electrochemical (ec)‐AFM, electrochemical strain microscopy (ESM),^34^, ^35^ hybrid AFM‐SECM^36^, ^37^ have been used to characterise interfaces, formed interphases, nanomechanical properties and strain of electrodes used in LIBs and post‐Li‐ion batteries.^38^, ^39^, ^40^, ^41^, ^42^, ^43^ For example, AFM was employed to study the SEI's morphology and mechanical properties such as the Young's modulus, on various materials including HOPG,^44^ micro‐sized Si,^45^ graphite^46^ or HC.^39^ Among the AFM‐based techniques, ESM is highly suitable, as the AFM tip, which is biased with an AC potential at high frequency, induces ion concentration gradients within the volume below the AFM tip leading to electrochemical strain and changes in surface height (Figure 1A). To avoid the screening of the tip potential, the technique is mostly conducted in the absence of electrolyte and not compatible with *operando* experiments. Without electrolyte, the tip acts as a selective contact for electron injection and extraction while ionic charges cannot be exchanged and are preserved in the electrode. The observed strain is therefore a consequence of ionic displacement, occurring as a consequence of oxidation and reduction of the electrode material below the AFM tip. As the strain is generated only in the interfacial volume fraction below the AFM tip, the ESM signal requires amplification by tip‐surface resonances at high frequencies (> 300 kHz) to be reliably measured.^34^ Differences in local electrochemical strain have been explained as a combination of changes in ion diffusivity, domain orientation and related changes in the Vegard‐coefficient. So far, ESM studies have been performed mainly for all‐solid‐state hybrid electrolytes and studies in liquid electrolyte solution are scarcely reported.^41^, ^47^, ^48^

In contrast, ec‐AFM (Figure 1B) maps the electrode´s surface morphology, for example, in electrochemical half‐cells to study SEI formation^49^ and Li^+^ insertion into graphite electrodes, as recently reviewed.^50^, ^51^ The ion insertion mechanism in HOPG was assessed by measuring the dimensional changes and correlating them to interlayer spacing variations associated with specific intercalation stages.^52^, ^53^, ^54^, ^55^ In the case of SIBs, Yang et al. recently emphasised how intercalation mechanisms and the resulting structural changes are directly linked to battery performance.^56^ However, tracking intercalation‐induced dimensional changes using ec‐AFM can be challenging due to SEI formation and other surface processes.^54^, ^57^ ec‐AFM measurements have shown that SEI layer formation on graphite anodes is influenced by suitable electrolyte additives, which lead to a harder and denser SEI layer suppressing more efficiently dendrite growth.^58^ Such denser SEI layers, with improved mechanical properties and higher charge transfer resistance for Li^+^‐ions, prevent the reduction and deposition of Li^+^‐ions, highlighting ec‐AFM as a powerful technique for studying the electrode/electrolyte interface and possible metallic‐dendrite growth. Recently, an advanced ec‐AFM technique was introduced that combines the electrochemical control of ec‐AFM with a small, superimposed AC voltage modulation, which results in a small modulation of the charge density of the locally probed electrode area. Such modulated electrochemical atomic force microscopy (mec‐AFM) was used to characterise ionic and electronic carrier dynamics in conducting polymer electrodes. In such mixed ionic electronic conductors, variation of charge density builds up a strong electrochemical strain due to electroswelling upon ion insertion.^59^ Technically, mec‐AFM experiments are the combination of ESM and ec‐AFM experiments, with the difference that the AC‐modulation is applied to the sample under investigation and the AFM probe is not electrically contacted but left floating. As such, the tip is sensitive to the surface morphology when operated in contact mode. In addition, the tip picks‐up the height modulation as caused by the AC‐signal when electrochemical strain is generated. By demodulation, a separate imaging signal is obtained that contains the amplitude and phase of the electrochemical strain signal. Critical for the mec‐AFM experiment is the RC time constant that determines the upper frequency limit up to which the surface charge of the electrode is modulated. For macroscopic thin films, the charging is limited to low frequencies by the large RC time constant associated to the large capacitance of the electrode and the electrolyte resistance. Instead, if micro‐sized samples are used, for example, microelectrodes, the RC time constant becomes small enough to permit charge modulation at kHz frequencies and by that efficient imaging of the electrochemical strain.^59^, ^60^

In this contribution, we explore mec‐AFM to study ion transport and related strain in HC composite anodes. To ensure high‐frequency surface charge modulation, we prepared micro‐sized HC composite electrodes by embedding the HC composite material in an epoxy polymer matrix. After polishing of the embedded sample, single well defined HC microparticles were obtained, which were then used in an electrochemical half‐cell configuration to perform in situ mec‐AFM measurements. Spectroscopic AFM experiments were carried out to investigate the bias, force, and frequency dependence of the electrochemical strain signal on different regions of the composite electrode. The findings of these studies point to the optimum conditions for electrochemical strain imaging. We further explored the multichannel mec‐AFM imaging to map strain at different electrode potentials. We demonstrate that in the initial stage of Na^+^ insertion, electrochemical strain primarily manifests in the softer binder matrix (consisting of the binder and the carbon black nanoparticles) rather than in the HC microparticles.

## 2 RESULTS

**FIGURE 2:**
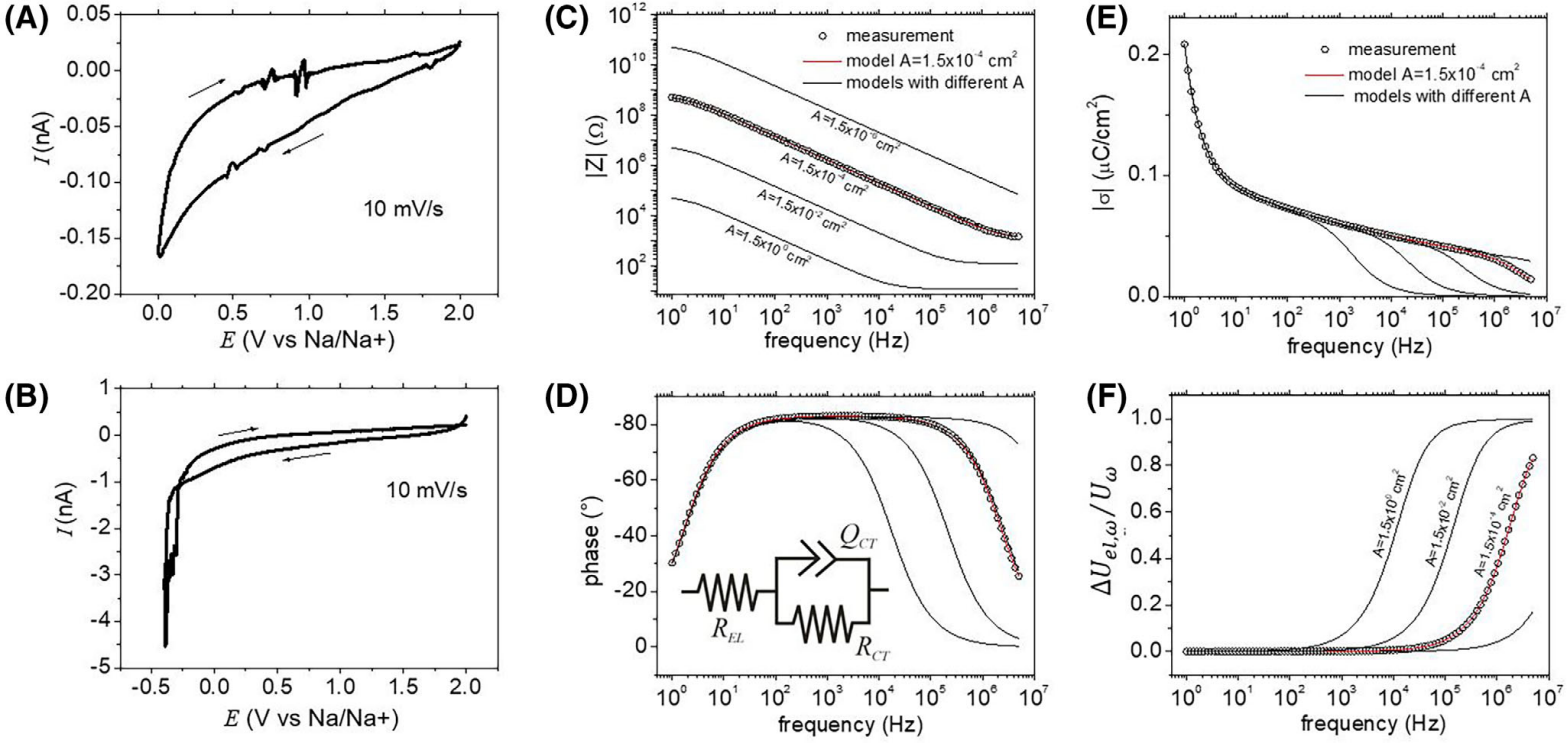
Electrochemical properties of the HC composite microelectrode (area *A* = 1.5 10^−4^ cm^2^): (A, B) Cyclic voltammograms recorded in two different potential ranges. At potentials < 0 V versus Na/Na^+^, electroplating of sodium occurred; (C, D) electrochemical impedance spectra magnitude and phase, (E) electrode surface charge density σ as a function of frequency; (F) normalised AC voltage drop $\Delta U_{el,\omega }/U_{\omega }$ in the electrolyte as a function of frequency. The inset in (D) shows the circuit model used to fit the electrochemical impedance data. To show the critical role of the electrode area *A*, we use the model with the fitted parameters to simulate electrodes with smaller or larger areas A as indicated in the graphs.

The inherent surface roughness and complex morphology of composite battery electrodes is a challenge for AFM studies. For model half‐cell measurements, the battery material can be embedded in epoxy resin,^39^, ^49^ which offers excellent chemical stability and compatibility with the commonly used electrolytes. Embedding HC composite electrodes and applying polishing steps results in a substantially reduced surface roughness of *S*_a_ = 0.06 ± 0.03 µm and *S*_z_ = 0.34 ± 0.18 µm (*n* = 5, 5.0 µm × 5.0 µm, line profiles in Figure S1). Figure 1C shows an optical microscope image of an embedded and polished HC composite electrode where. individual HC particles are visible. To reduce the electrochemical RC time constant, the electrode was covered with a fluoroelastomer coating and only a final part with an area of ca. 50 µm × 150 µm was left exposed to create the micro‐sized area, which is in contact with the electrolyte. The sample was then mounted into the electrochemical AFM cell as schematically shown in Figure 1D. Half‐cell experiments with sodium as the reference/counter electrode were performed in 1 M NaPF_6_ in ethylene carbonate/ propylene carbonate (EC/PC) with 5% fluoroethylene carbonate (FEC) as additive, which is a well‐studied reference electrolyte for SIBs.^61^ Under the specified measurement conditions, the measured electrode currents remained below 1 nA (Figure 2A), effectively ruling out any significant electrode polarisation. Only when polarising the electrode below 0 V versus Na/Na^+^ electroplating of sodium was observed (Figure 2B).

We conducted electrochemical impedance experiments (EIS) under blocking conditions (*E*  =  2.0 V vs. Na/Na^+^) to investigate the amount of ionic charge accumulation and transport at the HC microelectrode. The Bode plots of the impedance magnitude and phase are shown in Figure 2C and D, respectively, and the experimental data were fit to a Randles circuit in which the double layer capacitor is substituted by a constant phase element to account for small time constant dispersion. A more complex transmission line model, typically used to represent Na‐ion transport within the porous structure of HC,^62^ is not necessary for the epoxy‐embedded electrode. Fitting values for the circuit elements are: *R_CT_*  =  0.64 ± 0.05 GΩ; *Q_CT_*  =  (1.8 ± 0.3) × 10^−10^ Fs^α–1^; *α*  =  0.92 ± 0.2; *R_EL _* =  1.3 ± 0.1 kΩ. The large *R* *_CT_* value confirms the blocking conditions of the electrode. The constant phase element *Q_CT_* normalised per area is significantly smaller for the epoxy embedded electrode than values observed for non‐embedded HC composites^19^ and shows that ions accumulate only close to the interface with the electrolyte. Importantly for mec‐AFM measurements, the data demonstrates that for the investigated electrode with an area of ca. 1.5 × 10^−4^ cm^2^, the impedance is dominated over a wide frequency interval spanning from 20 Hz to 400 kHz by the capacitive charging of the electrode. Only at lower frequencies, resistive charge transfer processes at the HC interface start to dominate. Above 400 kHz the electrolyte resistance becomes relevant. For mec‐AFM experiments, a broad frequency interval with capacitive charging is important, as the signal is generated by the ionic charge modulation occurring during each AC cycle at external or internal interfaces of the electrode. To illustrate this concept in more detail, we compare our impedance measurement data to simulated impedance curves for electrodes with areas varying over several orders of magnitude as shown in Figure 2B and C. For these simulations, we scale *R_CT_* and *Q* with the electrode area (*R_CT_* ∼ 1/*A* and *Q_CT_* ∼ *A*). Instead for the electrolyte resistance, we used a scaling of *R_EL_* ∼ $1/\sqrt{A}$ following the equation for square‐shaped microelectrodes: $R_{EL}=\rho \ln4/\pi \sqrt{A}$ in which ρ is the electrolyte resistivity (ρ=35Ωcm). Therefore, with increasing electrode size, the electrolyte resistance starts to dominate the impedance at lower frequencies. From the impedance $Z_{\omega }$ we can also directly calculate the charge density modulation $|\sigma_{\omega }|=U_{\omega }/A\omega \left|Z_{\omega }\right|$ of the HC electrode and the voltage drop $\Delta V/V_{\omega }$ of the AC signal in the electrolyte. The resulting frequency dependencies of $\sigma_{\omega }$ and $\Delta U/U_{\omega }$ are shown in Figure 2E and F. From this data, we obtain that only micro‐sized electrode areas enable a wide plateau with almost constant surface charge modulation *σ*. For our epoxy‐embedded microelectrode, we achieve $\sigma_{\omega }$ = 0.07 µC/cm^2^. We note that the small drop in charge modulation with frequency is caused by the time constant dispersion as described with the constant phase element. Complementary, Figure 2F reveals that when the frequencies become too large, the AC signal no longer dropped over the solid/electrolyte interface but decays in the electrolyte, and the charge modulation becomes negligible. Overall, the data illustrates why micro‐sized electrode dimensions are required to achieve high charge density modulation at the solid/electrolyte interface over a wide frequency interval, which is required for mec‐AFM imaging.

**FIGURE 3:**
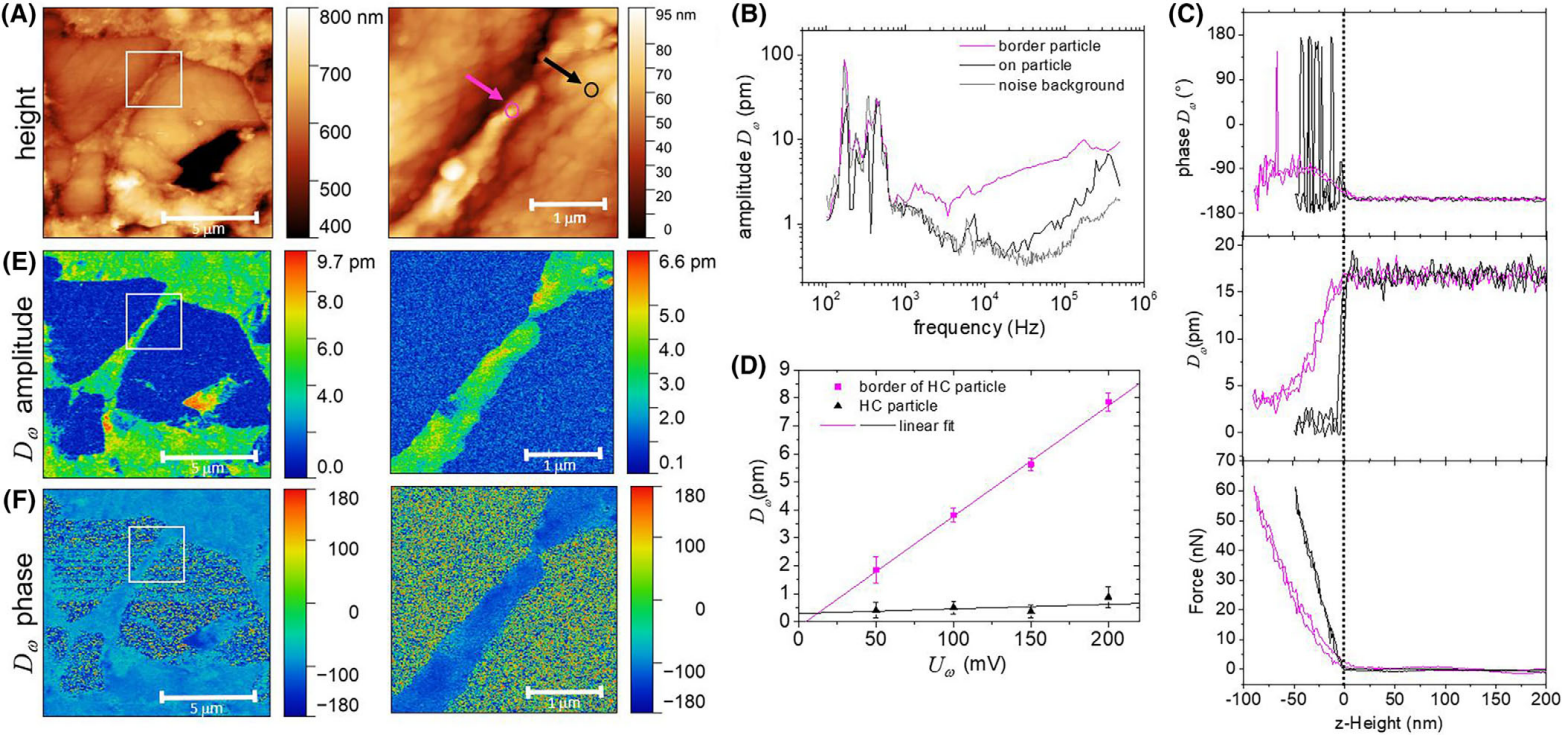
Investigation of HC particles with mec‐AFM at a blocking potential (*E* = 2.0 V vs. Na/Na^+^). (A) Height image. The white rectangle represents the measurement area of the zoomed image. The purple and black arrows point to the pixels where detailed spectroscopic investigations were conducted. (B) Frequency spectrum of the mec‐AFM electrochemical strain signal *D_ω_* recorded at two different positions. Also, the noise background is shown. AFM spectroscopy data showing the force and the mec‐AFM channel amplitude *D_ω_* and phase as a function of z‐scanner height during approach and retraction. (C) AFM spectroscopy data showing the force and electrochemical strain amplitude *D_ω_* and phase as a function of z‐scanner height during approach and retraction. (D) Electrochemical strain amplitude *D_ω_* as a function of the driving amplitude *U_ω_* as measured on the two different surface positions. (E, F) Electrochemical strain amplitude *D_ω_* and phase images. If not otherwise stated, measurements were conducted at *f* = 67 kHz, *U_ω_* = 100 mV and *F_set_* = 35 nN.

After these studies, AFM imaging experiments were conducted in contact mode using a cantilever with a nominal force constant of *k*  =  6 N/m. In this initial phase of the experiment, all measurements were carried out applying *E*  =  2.0 V versus Na/Na^+^ to the electrode (blocking conditions). Images of measured height maps are shown in Figure 3A. The presence of HC microparticles can clearly be distinguished as flat, homogeneous surfaces with a clear border. The space in between the HC particles appears less homogeneous, with a more granular surface that is associated with the binder and black carbon nanoparticles. In the following, we call this phase the matrix of the electrode into which HC microparticles are embedded (see also SEM cross section in Figure 1). To investigate surface interactions related to ion transport in this regime, we selected two characteristic regions, one on the HC microparticle and the other one located on the gap between two microparticles as indicated in the AFM height map. While keeping the tip at these positions, we applied an additional AC signal with an amplitude $U_{\omega }$ = 100 mV to the electrode and swept the frequency while we recorded the resulting cantilever vibration amplitude $D_{\omega }$ as shown in Figure 3B. We compared the frequency sweeps obtained at the two different positions and analysed also a measurement of the background noise. Up to 1 kHz, all three signals are similar and show oscillations that exceed several 10 pm in amplitude. These oscillations are attributed to the noise background of the glovebox environment and are not further considered. Above 1 kHz, a clear signal emerged for the measurement done on the binder/carbon matrix surface in close proximity to the HC particle. This signal maintained a good signal to noise ratio up to 500 kHz. Instead, on the HC particle surface only a small signal appeared that exceeded the background noise at frequencies above 40 kHz. Based on these findings, we used a modulation frequency of 67 kHz for the following experiments.

Next, we conducted force distance spectroscopy experiments while the AC modulation was kept constant at 67 kHz and $U_{\omega }$ = 100 mV at the two different surface positions. The resulting force curves along with the tip oscillation amplitude *D_ω_* and phase as a function of tip height are shown in Figure 3C. Prior to the contact of the tip with the surface, a tip oscillation is present with an amplitude of approx. 17 pm, independent on the chosen position. This oscillation is associated to the ionic AC current and the related electric field present in the electrolyte, causing a residual force acting on the AFM tip. Upon contact with the surface, the modulation diminished and on the more rigid HC surface, *D_ω_* dropped to zero. On the binder/black carbon matrix surface in close vicinity to the HC particle, the force curve showed a more elastic response behaviour, which is associated with the softer mechanical properties of the matrix compared to the HC particle. Upon contact, the tip oscillation *D_ω_* was also reduced and followed the contact force. However, at larger repulsive forces, the tip oscillation showed a plateau value of $D_{\omega }$ =  4 pm that was no longer dependent on the magnitude of the repulsive force. At these larger forces also the phase value stabilised at –94°. This behaviour demonstrates the transition from electric field to electrochemical strain‐induced tip oscillations on the binder/ black carbon matrix area. In Figure 3D, the linear scaling of the oscillation signal $D_{\omega }$ with the driving amplitude $U_{\omega }$ is shown when measured in contact with the surface. Overall, these findings indicate that an electrochemical strain signal caused by ions entering into the binder/black carbon matrix, can be revealed when the force setpoint is kept sufficiently high to avoid contributions due to the electric field. Instead on the HC surface, even at higher $U_{\omega },$ no significant signal emerges demonstrating the absence of electrochemical strain.

With this understanding about how the signals are generated, we set the parameters for the mapping of the oscillating electrochemical strain to ω = 67 kHz, $U_{\omega }$ = 100 mV and set point force *F* = 35 nN. Using these settings during contact mode imaging, a value for the amplitude and phase of $D_{\omega }$ was obtained for each pixel. The resulting maps are shown in Figure 3E and F. A clear contrast is visible between the HC microparticle surface and the surrounding matrix containing binder and carbon black nanoparticles. On the HC particles, no electrochemical strain is detected. The amplitude $D_{\omega }$ was within the noise level of the measurement and the phase had random values. Instead on the matrix surface, we observed stable surface height oscillations $D_{\omega }$ with an amplitude ranging between 3 and 10 pm and a phase value of ca. −90°. Enhanced strain is localised at the interface between the HC particle and the surrounding matrix (Figure 3A). A scan performed in a smaller region with a scan length of 3 µm demonstrates the excellent resolution of the electrochemical strain mapping. In the observed region, a small gap of some tens of nanometres of width between two HC microparticles became visible in the electrochemical strain signal with a clearly observable contrast.

The combined measurement of the electrochemical strain image and the electrochemical impedance spectroscopy allow to do a simple quantitative analysis to estimate the effective radius of Na‐ions *r_Na_* entering the matrix containing binder and carbon black nanoparticles. The strain image shows that only the matrix surface regions contribute to the strain and the average amplitude is *D_ω_* = 5.1 ± 0.8 pm (see Figure S3). The ratio of matrix surface $A_{b}$ to total area A is $A_{b}/A=0.1$. Assuming an uncompressable behaviour, we can compute the increase in volume $\Delta V_{\omega }$ to be $V_{\omega }=D_{\omega }\;A_{b}$. The volume contributed by a single Na‐ion $V_{Na}$ is then $V_{Na}=\Delta V_{\omega }q_{0}/\sigma_{\omega }A$ where $q_{0}$ is the elementary charge and $\sigma_{\omega }$ is the surface charge density at 67 kHz as determined above ($\sigma_{\omega }$= 0.05 µC/cm^2^). Based on these estimations, we obtain $r_{Na}={\left(3V_{Na}/4\pi \right)}^{1/3}$= 330 ± 60 pm. The value is a factor of 3 larger than the pure radius of the Na‐ion and is in the range of typical cationic solvent radii. From these estimations, we hypothesise that the strain is generated by solvent molecules entering with the ions into the binder/carbon black matrix during Na+ insertion.

**FIGURE 4:**
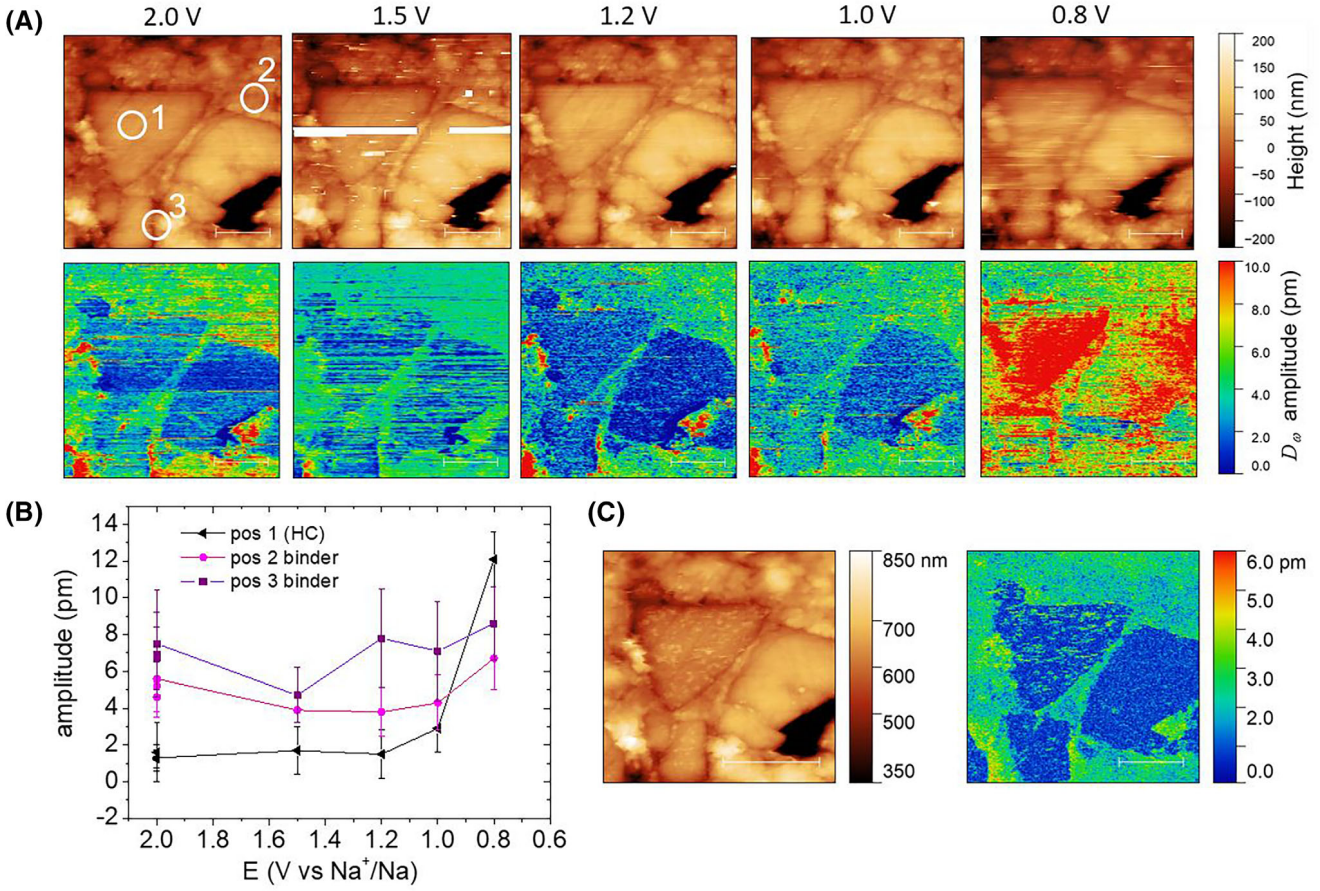
(A) mec‐AFM images of height and electrochemical strain amplitude *D_ω_* recorded at different potentials *E* applied to the working electrode. (B) Average values and standard deviation of *D_ω_* at three different regions as indicated with white circles in (A). Position 1 corresponds to the surface of a HC particle. Position 2 and Position 3 are located on the matrix surface close to the border of HC particles. (C) mec‐AFM scan (at *E* = 2.0 V vs. Na/Na^+^) obtained after cycling the voltage down to *E* = 0 V versus Na/Na^+^. The HC particle surface is covered by residues that exhibit a signal in the electrochemical strain channel. Successive scanning in contact mode removes the residues, which are attributed to SEI formation.

Next, we investigated how the electrode surface evolves when Na^+^ insertion starts to occur. For this purpose, we acquired mec‐AFM images at progressively smaller potentials applied to the electrode. Figure 4 shows the resulting height and electrochemical strain maps. Due to a faster scan velocity, the strain signal contains more noise than in the maps shown in Figure 3, but one can still clearly distinguish HC particles from matrix regions. As before, areas with increased electrochemical strain are present on the matrix surface in close proximity to HC particles. No strain signal was observed on the surface of the HC particles. When decreasing the applied potential, the observations remained stable until the applied potential was below *E* = 1.0 V versus Na/Na^+^. At that point, the electrochemical strain signal started to increase, initially exhibiting a slight rise in the matrix area, followed by a more pronounced increase on the HC surface at *E* = 0.8 V versus Na/Na⁺. We suggest that SEI formation led to increasingly unstable AFM tip surface interactions, which affected also the morphology measured in contact mode. The potential region below 1.0 V versus Na/Na^+^ is characterised by Na^+^ insertion (so‐called sloping region) where a strong dependence of the capacity with the applied potential is observed.^29^, ^30^ In this potential range, also the formation of the SEI occurs due the reduction of electrolyte species.^40^ We continued to cycle the electrode to lower potentials, but stable artefact‐free, contact mode images could not be reliably acquired. When turning back to blocking potential conditions, we observed the deposition of islands of material on the HC‐surface, which we associate with SEI formation. These islands show also a contrast in the electrochemical strain signal (Figure 4C). However, with continuous contact mode scanning, we observed that the islands were removed from the HC surface (see Figure S4) and thus do not permit a reliable characterisation in contact mode.

Although limited in the potential range, the *operando* mec‐AFM characterisation provided information about the initial phase of Na^+^ adsorption at defect sites and Na^+^ insertion that occurs prior to the formation of a SEI layer. From battery charging experiments, it is well known that in the initial phase of Na^+^ insertion, the stored charge is strongly dependent on the potential (‘sloping region in the potential capacity curve’).^16^, ^27^, ^29^ In this initial charging phase, our measurements showed no significant electrochemical strain on the HC particles, which we associate with no volume expansion occurring in the HC microparticles in this potential range. We interpret this finding with Na^+^ adsorption at defect sites, or entering only into nanopores filled with electrolyte or other interfacial HC regions in contact with electrolyte. Such a process corresponds more to an adsorption process, as it does not involve any distortions of the carbon lattice structure. On the binder matrix/carbon black area, the observed response showed a small volume expansion. The nanomechanical properties of the binder/black carbon matrix are less stiff and more permeable to ions and electrolyte. The potential variation then causes a pseudo‐capacitive charging with ions entering into the binder/carbon black matrix with their surrounding solvent shell. We hypothesise that the associated volume expansion in the binder/carbon black matrix is the cause for the small electrochemical strain signal observed in our experiments.

## 3 CONCLUSIONS

In conclusion, our study investigated the potential of mec‐AFM to investigate and visualise Na‐ion insertion and electrochemical strain in HC composite electrodes. For mixed ionic electronic conductors based on conducting polymers, mec‐AFM was demonstrated to be a reliable method to achieve quantitative mapping of the electrochemical strain that builds‐up due to ion insertion.^59^, ^60^ Here, we show how mec‐AFM experiments can be performed on HC‐based composite electrodes used for SIBs. Our findings show that also for this case, the additional AC modulation results in a novel imaging channel that provides contrast based on ion insertion and enables to distinguish different material regions on the electrode. Regions containing the binder and carbon black nanoparticle matrix are distinguished by a clear electrochemical strain signal. Instead on the HC surface, no strain related signal could be detected. We explain this contrast by differences in ion insertion, where Na‐ions with solvent shell enter into the binder/nanoparticle matrix causing a small volume expansion. Due to the investigations performed at high, blocking potentials (2.0 – 1.2 V versus Na/Na^+^), only minor amounts of Na‐ions enter into HC microparticles and occupy adsorption sides, that are not related to distortions of the carbon lattice. Our work shows that a micrometre‐sized electrode area is critical to perform measurements at higher frequencies with better signal‐to noise ratio in the strain signal.

As a remaining challenge in our approach, we identified the relatively small charge density modulation observed within the tested potential range. Since only a limited number of ion exchanges related to the AC signal occur, the corresponding electrochemical strain signal remains modest, reaching a maximum of a few tens of picometres. Such small signals pose challenges in distinguishing them from the noise background or oscillations due to electric fields in the electrolyte. Instead, at lower half‐cell potentials where larger amounts of ion insertion are expected to happen, measurements become instable due to the SEI formation occurring at 1.0 to 0.8 V versus Na/Na^+^. In contact mode, we observed that the tip removes the formed SEI during scans, leading to instability in the interaction under in situ conditions. For future measurements, we see potential for imaging approaches that combine mec‐AFM with Pin‐Point or Force–Volume approaches avoiding the constant tip/sample contact with shear forces inducing SEI instabilities. By performing measurements at higher charge capacities, we expect to see more significant electrochemical strain signals associated with the formation of metallic clusters inside HC nanopores.^29^, ^30^

## 4 METHODS

### 4.1 Reagents and materials

Propylene carbonate (PC) and Na‐CMC were obtained from Sigma Aldrich, Germany. Sodium was obtained from Alfa Aesar, Germany. HC was obtained from Kuraray (Kuranode type 2, 5 µm, Japan). Carbon black was purchased from Cabot Corporation (Vulcan XC72R, USA).

### 4.2 Sample preparation and characterisation

The HC particles were mixed with CC and Na‐CMC in a mass ratio of 85:10:5, stirred at 50°C for 48 h in a mixture of isopropyl alcohol and water (1:1). The resulting slurry was spray‐coated onto a PET substrate and subsequently dried at 80°C for 30 min. During the drying process, the PET support sheet was delaminated. A small section of the dried composite electrode was embedded in epoxy resin (EpoFix, Struers GmbH, Germany) following a vacuum embedding procedure, with subsequent hardening at room temperature, grinding, polishing and electrically contacting as described elsewhere.^39^ The polishing quality was assessed with a VHX‐7000 digital microscope (Keyence, Japan). To achieve the desired electrode size of 50 µm × 150 µm, the sample was insulated with a FFKM‐based layer (3 M Belgium BVBA).

### 4.3 Electrochemical experiments

All measurements were conducted inside a glovebox (Unilab, MBraun, Germany) under an argon atmosphere, with oxygen and water levels below 0.1 ppm. Electrochemical cycling tests were carried out using an electrolyte with 1 mol/L NaPF_6_ in EC/PC and 5% v/v FEC. The embedded HC anode served as the working electrode and sodium was used as reference/counter electrode, connected to a bipotentiostat (CHI760E, CH Instruments). Cyclic voltammetry was performed at a scan rate of 2–10 mV/s in a potential range of 0.01–2.0 V versus Na/Na^+^. Electrochemical impedance spectroscopy measurements were performed with a MFLI Lockin Amplifier (Zurich Instruments) with a modulation amplitude of 10 mV.

### 4.4 AFM and force spectroscopy

The AFM (Park NX10, Park Systems, South Korea) is located in the glovebox. Imaging in contact mode and force spectroscopy were conducted at forces *F* ∼ 35 nN, using etched silicon probes (RTESPAW‐150, Bruker, Germany) with a nominal spring constant of *k* = 6.0 N/m and a resonance frequency of 150 kHz at a scan speed of 0.2 Hz. Prior to measurements, the force constants of the cantilevers were determined using the thermal noise method.^63^ Force–distance curves were acquired at a sweep rate of 0.1 µm/s with loading forces of 60 nN. AFM images (12.0 × 12.0 µm, 256 × 256 pixels and 3.0 × 3.0 µm, 256 × 256 pixels) and force–distance curves were analysed using Gwyddion (version 2.65). The roughness parameters of the polished sample were obtained in contact mode AFM (imaged area: 12 × 12 µm, 256 × 256 pixel, five 5 × 5 µm areas). The mec‐AFM method was implemented with an external MFLI lock‐in amplifier (Zurich Instruments). The signal output was a generated sine wave with controlled amplitude *U_ω_* and offset voltage *E* that was applied to the working electrode. As signal input we connected the amplified PSPD signal from the AFM. The sweeper functionality of the MFLI‐software was used to perform measurements as a function of signal frequency and amplitude. The lockin amplifier time constant was automatically adjusted using the impedance sweep functionality (sweep rate slow/high precision). For imaging, the modulus and phase of the demodulated signal were amplified and connected to the Aux2 and Aux3 input of the AFM and added as additional imaging channels. During imaging, we used a time constant of τ = 4 ms and a filter order *n* = 8. To translate the signal into the oscillation amplitude *D*_w_, we measured the sensitivity of the AFM probe with a force distance spectroscopy on a solid substrate (*S* = 52 V/µm) and multiplied with the lock in signal gain (*G* = 4000)

### 4.5 FIB/SEM

Before cross‐sectioning the embedded HC composite electrodes, a thin Pt layer was sputtered onto the sample to reduce charging effects and to protect the sample from ion beam damage. A protective platinum layer of approx. 300 nm was deposited using ion beam‐induced deposition (IBID) with methylcyclopentadienyl trimethyl platinum (C_9_H_16_Pt) as precursor. Cross‐sectioning and imaging was carried out using a dual beam system (Helios NanoLab 600, ThermoFisher Scientific, USA).

## Supporting information



Supporting Information

## Data Availability

The data that fort the findings of this study are openly available at Zenodo https://doi.org/10.5281/zenodo.14764314, reference number 441.
