# Supplementary material for: Modulated electrochemical force microscopy: Investigation of sodium‐ion transport at hard carbon composite anodes
Source: J Microsc. 2025 May 9;302(1):17–28. doi: 10.1111/jmi.13417 (PMC13045773; doi:10.1111/jmi.13417)
Supplement: Supplementary file 1 — Supporting Information [file JMI-302-17-s001.docx]

**Supporting Information**

**Modulated Electrochemical Force Microscopy: Investigation of Sodium-Ion Transport at Hard Carbon Anode Surfaces**

S. Daboss,^[a]^ N. Franke,^[b]^ B. Fraboni,^[b]^ C. Kranz,^[a]^ T. Cramer^[b]^

[a] Dr. Sven Daboss, Prof. Dr. Christine Kranz
Institute of Analytical and Bioanalytical Chemistry (IABC)
Ulm University, Albert-Einstein-Allee 11, 89081 Ulm (Germany)

[b] Nikolas Franke, Prof. Beatrice Fraboni, Prof. Dr. Tobias Cramer

Department of Physics and Astronomy

University of Bologna, Viale Berti Pichat 6/2, 40127 Bologna (Italy)

E-mail: [tobias.cramer@unibo.it](mailto:tobias.cramer@unibo.it)


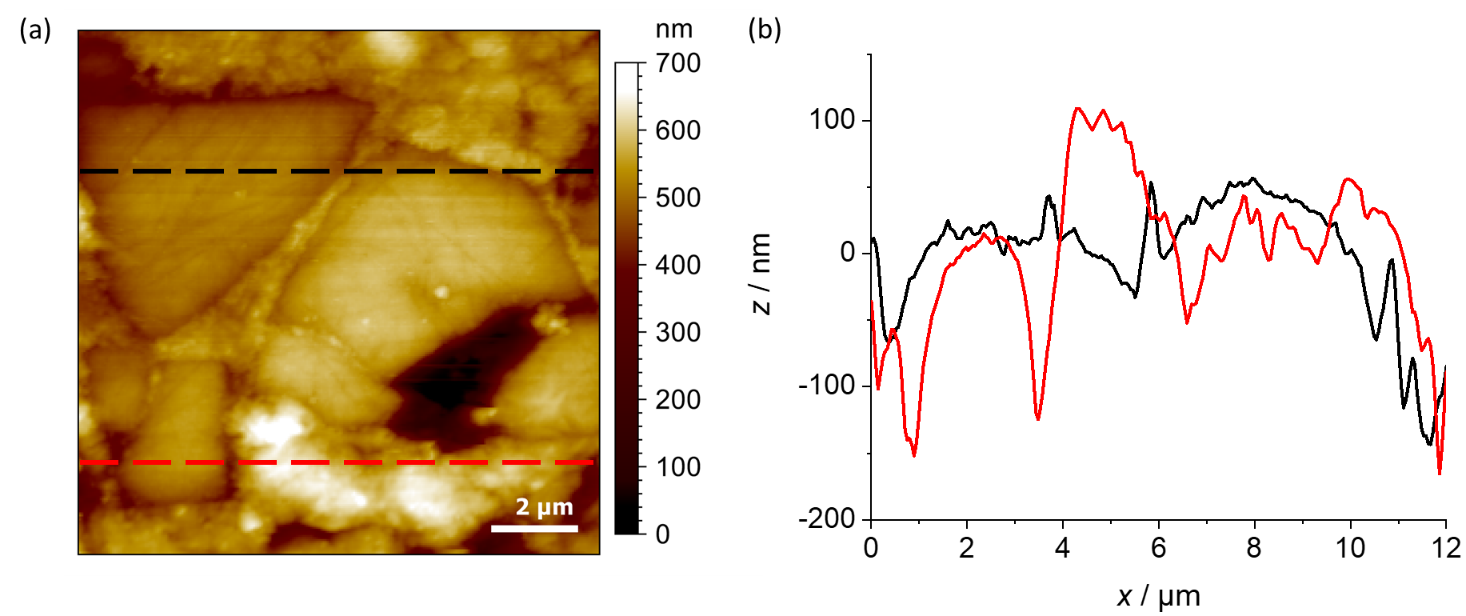


**Figure S1:** (a) AFM topography image of the embedded HC composite anode. (b) Line scans extracted as marked in the images with the black dotted line and the red dotted line.


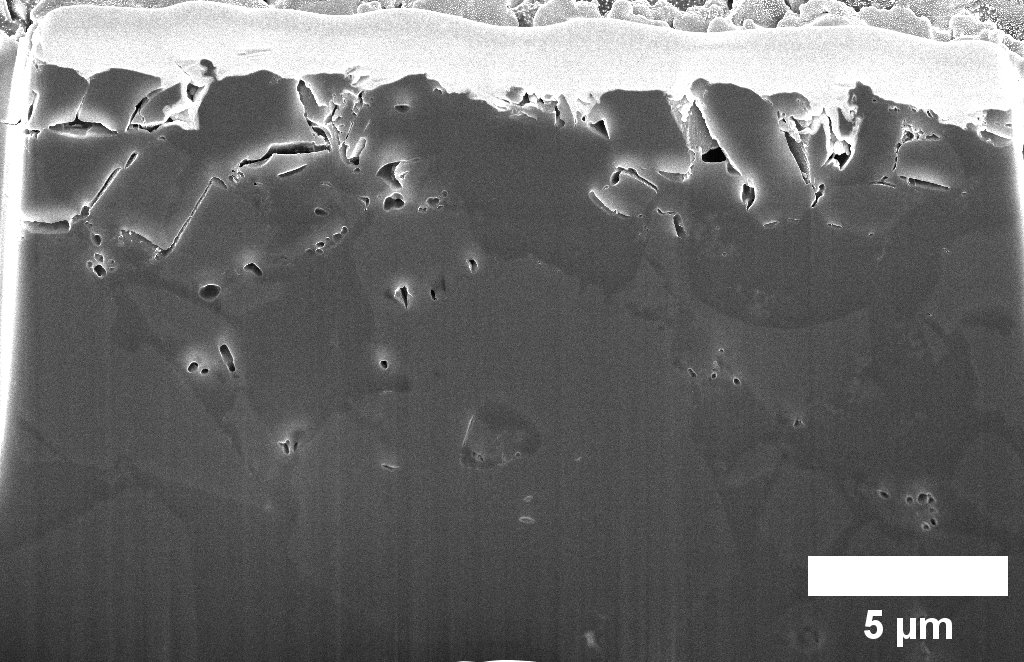


**Figure S2:** FIB cross-section of the embedded HC composite electrode. The image reveals the electrode architecture, size and the distribution of active material.

**Figure S3:** Analysis of AFM images of Figure 3e – distribution of |*D_ω_*| and fit to Gaussian function to obtain an average value.


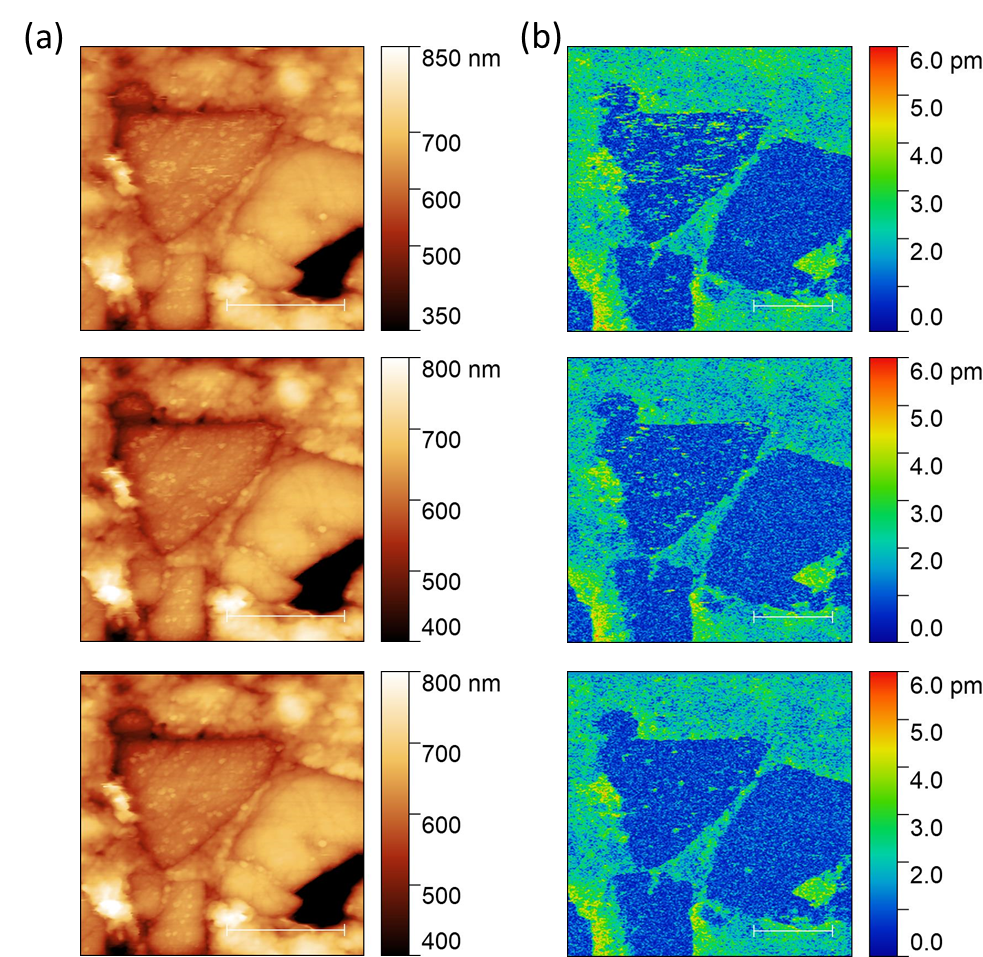


**Figure S4:** (a) mec-AFM images (*E* = 2.0 V versus Na/Na^+^) of height and (b) surface oscillation amplitude *D_w_* showing accumulation of material on the HC particle and their partial removal with successive scanning.
